# Efficient Routes to Pyrazolo[3,4-*e*][1,2,4]triazines and a New Ring System: [1,2,4]Triazino[5,6-*d*][1,2,3]triazines

**DOI:** 10.3390/molecules15053302

**Published:** 2010-05-04

**Authors:** Hamad Mohamed Al-Matar, Khaled Dessouky Khalil, Doa’a Mohamed Al-Dorri, Mohamed Helmy Elnagdi

**Affiliations:** 1 Chemistry Department, Faculty of Science, University of Kuwait, P.O. Box 5969, Safat 13060, Kuwait; E-Mail: shelmy1941@yahoo.com (M.H.E.); 2 Chemistry Department, Faculty of Science, Cairo University, Giza 12613, Egypt; E-Mail: khd.khalil@yahoo.com (K.D.K.)

**Keywords:** amidrazones, arylhydrazononitrile, amidoximes, [1,2,4]triazino[1,2,3]triazine, dimethylformamide, dimethylacetal, pyrazolo[1,2,4]triazine

## Abstract

Arylhydrazonomalononitriles **1a,b** react with phenylhydrazine to yield amidrazones **2a,b** that cyclize to give 2-aryl-5-phenylhydrazono-2,5-dihydro-[1,2,4]-triazine-6-carbonitriles **5a,b** upon reaction with dimethylformamide dimethylacetal (DMFDMA). Refluxing **5a,b** in glacial acetic acid resulted in the formation of the pyrazolo-1,2,4-triazines **6a,b**. Compounds **6a,b** were also formed upon treatment of 3-amino-4-phenylhydrazono-1-phenyl-2-pyrazolin-5-ones **7a,b** with DMFDMA. Reacting these triazinyl arylhydrazononitriles **5a,b** with hydroxylamine hydrochloride in ethanolic sodium acetate afforded amidrazones **8a,b** that are readily cyclized in refluxing dimethylformamide into [1,2,4]triazino[1,2,3]triazines **10a,b**.

## Introduction

**Scheme 1:**
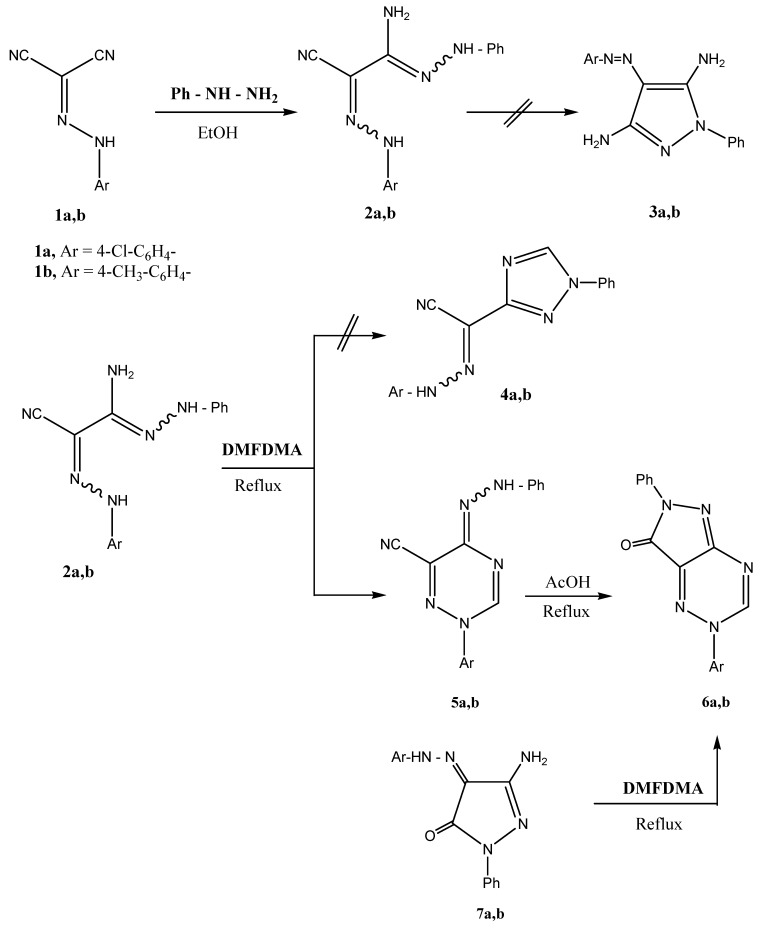
Proposed mechanism for the formation of 2-aryl-6-phenyl-2*H*-pyrazolo[3,4-e][1,2,4]triazin-7(6*H*)-ones **6a,b**.

Arylhydrazonomalononitriles **1** are synthetically useful reagents that have been utilized in the past as precursors to 4-arylazo-3,5-pyrazolediamines as well as [1,2,3]triazoleamines and pyrazolo[1,5-*a*]pyrimidines [1]. While reaction of **1** with hydrazine hydrate is established to yield pyrazolediamines that have been patented as a hair dyes, similar treatment with hydroxylamine hydrochloride has resulted, in our hands, in isolation of amidoximes that were utilized as precursors to other heterocycles [2,3]. It occurred to us that there might be value in trying to isolate amidrazones from the reactions of **1a,b** with substituted hyrazines [4,5,6,7]. We report here the results of our reinvestigation of the behavior of compounds **1a,b** toward phenylhydrazine. 1,2,3-Triazine derivatives are an important class of heterocyclic compounds useful in organic synthesis and as pharmaceuticals (e.g., as antimalarials) [8,9,10]. The work enabled development of an easy route to pyrazolo[3,4-*e*][1,2,4]triazines and [1,2,4]triazino[5,6-*d*][1,2,3]triazines (a new ring system) (Scheme 1) [11]. 

## Results and Discussion

Reacting 2-arylhydrazonomalononitriles **1a,b** with phenylhydrazine at room temperature in ethanol afforded 3-amino-2-arylhydrazono-3-phenylhydrazonopropanenitriles **2a,b**. This is in contrast to the reported formation of **3a,b** upon extended refluxing of **1a,b** and phenylhydrazine in ethanol (Scheme 1) [3]. Although **2a,b** may also exist in other tautomeric forms, only **2a,b** were produced, as indicated from the ^1^H-NMR spectra that revealed D_2_O exchangeable amino signals at δ 6.09, 4.03 ppm and two hydrazone NH signals at δ 8.87, 9.67 and δ 11.10, 12.48 ppm, respectively. Compounds **2a,b**, so formed, reacted readily and smoothly with dimethylformamide dimethylacetal (DMFDMA) to yield the products of condensation with elimination of two molecules of methanol and one molecule of dimethylamine. These can thus be formulated as the 2-aryl-5-phenylhydrazono-1,2,4-triazines **5a,b** or the isomeric 1,2,4-triazoles **4a,b**. Structures **5a,b** could be established for these products based on the ^1^H-NMR spectra which revealed characteristic C-H signals at δ 9.67 and 8.76 ppm, respectively. If the products were **4a,b** these signals should have appeared at higher field (δ ~ 8 ppm) [12]. Moreover the reaction product **5a,b** cyclized upon refluxing in acetic acid to yield **6a,b**. Heterocyclic ring systems similar to **6a,b** were prepared by reacting **7** with acetic anhydride to give the methyl derivatives of **6** [13]. If the reaction products were **4a,b** they should be recovered unreacted under these conditions. Compounds **6a,b** could also be obtained via reaction of **7a,b** with (DMFDMA) (*cf*. Scheme 1). 

**Scheme 2:**
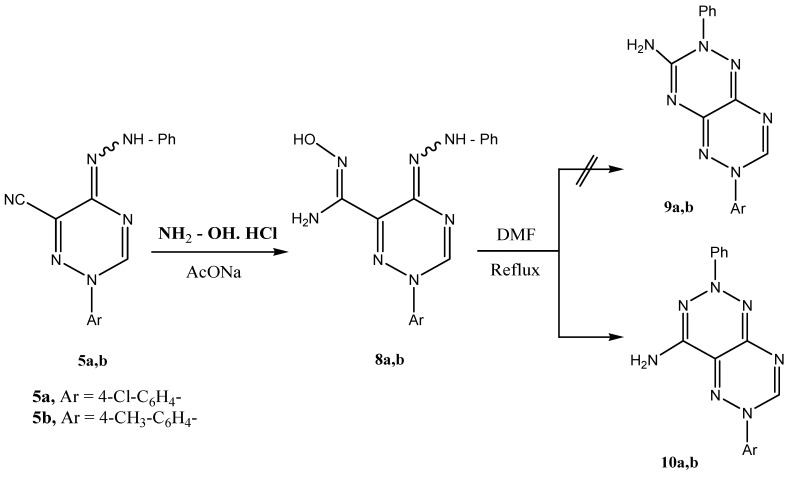
Proposed mechanism for the formation of 6-aryl-2-phenyl-2,6-dihydro-[1,2,4]triazino[5,6-d][1,2,3]triazin-4-amines **10a,b**.

Compounds **5a,b** reacted with hydroxylamine hydrochloride in ethanolic sodium acetate to yield 1,2,4-triazine-6-carboximidamides **8a,b**. Refluxing the latter in dimethylformamide (DMF) resulted in cyclization via water elimination to yield the [1,2,4]triazino[5,6-*d*][1,2,3]triazines **10a,b** (Scheme 2). 

Possible Tiemann rearrangement of **8a,b** prior to cyclization as reported earlier [14] for similar systems could be discounted as irradiation of the NH_2_ signal at δ 6.12 ppm did not enhance the integration of the aromatic multiplet at δ 7.44–8.02 ppm, as would be the case if the products were **9a,b**. HMBC ^15^N-NMR indicated the amino protons at δ 6.14 ppm to have a ^4^*J* cross peak with the sp^2^ nitrogen at δ 280 ppm. A Tiemann rearrangement product should show such a cross peak with the sp^3^ nitrogen at δ 230 ppm (*cf*. Scheme 2).

## Experimental

### General procedures

Melting points were recorded on Gallenkamp apparatus and are uncorrected. Infrared spectra (KBr) were determined on a Perkin-Elmer 2000 FT-IR system. NMR measurements were determined on a Bruker DPX spectrometer, at 600 MHz for ^1^H-NMR and 125 MHz for ^13^C-NMR, in DMSO-*d*_6_ as solvent and using TMS as internal standard. Mass spectra were measured on MS 30 and MS 9 (AEI) spectrometers, with EI 70 eV. Elemental analyses were performed by using of LECO CHNS-932 Elemental Analyzer. Copies of the original data can be provided upon request. 

### Synthesis of arylhydrazonomalononitriles ***1a,b***

These compounds were prepared following the literature procedure [6]. A cold solution of aryldiazonium salt (10 mmol) was prepared by adding a sodium nitrite solution (1.4 g dissolved in 10 mL water) to a pre-cooled solution of arylamine hydrochloride (10 mmol of arylamine in 6 mL of 6 M HCl) with continuous stirring. The resulting solution of aryldiazonium salts were then added carefully to a cold ethanolic solution (50 mL) of malononitrile (0.66 g, 10 mmol) and sodium acetate trihydrate (2.8 g, 20 mmol). The mixture was stirred at room temperature for 1 h and the solid product formed was collected by filtration, washed with water and recrystallized from ethanol.

*2-p-Chlorophenylhydrazonomalononitrile* (**1a**). Yellow solid (83%), m.p. 186–188 °C (as reported in literature [15]); IR: *υ* = 3260 (NH), 2188.4 (CN), 2198 (CN) cm^−1^; ^1^H-NMR: *δ* = 7.26 (d, 2H, *J* = 8 *Hz*), 7.93 (d, 2H, *J* = 8 *Hz*), 13.08 (s, 1H, NH); ^13^C-NMR: *δ* = 117.8 (2 CN), 121.1, 123.6, 130.4, 139.8 (aromatic carbons), 159.2 (C(CN)_2_); MS, *m*/*z* (%), 204.0 (M^+^, 100), 126 (62), 111.0 (77); Anal. Calcd. for C_9_H_5_ClN_4_: C, 52.83; H, 2.46; Cl, 17.33; N, 27.38. Found: C, 52.80; H, 2.41; Cl, 17.28; N, 26.32. 

*2-p-Tolylhydrazonomalononitrile* (**1b**). Yellow solid (74%), m.p. 169–170 °C (as reported in literature [16]); IR: *υ* = 3240 (NH), 2185.1 (CN), 2187 (CN) cm^−1^; ^1^H-NMR: *δ* = 2.6 (s, 3H, CH_3_), 7.02 (d, 2H, *J* = 8 *Hz*), 7.74 (d, 2H, *J* = 8 *Hz*), 12.6 (s, 1H, NH); ^13^C-NMR: *δ* = 23.6 (CH_3_), 117.4 (2 CN), 119.5, 125.3, 128.0, 135.9 (aromatic carbons), 157.6 (C(CN)_2_); MS, *m*/*z* (%), 184.1 (M^+^, 100), 91.1 (66); Anal. Calcd. for C_10_H_8_N_4_: C, 65.21; H, 4.38; N, 30.42. Found: C, 65.18; H, 4.32; N, 30.37.

### Reaction of ***1a,b*** with phenylhydrazine 

A mixture of arylhydrazonomalononitrile **1a,b** (10 mmol) and phenylhydrazine (1.08 g, 10 mmol) was dissolved in ethanol (50 mL) and the reaction progress was followed by TLC till completion after 24 h. The reaction mixture was cooled and the solid product, so formed, was then collected by filtration and recrystallized from ethanol. 

*3-Amino-2-p-chlorophenylhydrazono-3-phenylhydrazonopropanenitrile* (**2a**). Yellow solid (71%), m.p. 133–135 °C; IR: *υ* = 3244 (br., NH_2_ and NH groups), 2223.5 (CN), 1632, 1624 (two C=N) cm^−1^; ^1^H-NMR: *δ* = 6.09 (s, 2H, NH_2_), 7.12 (d, 2H, *J* = 8 *Hz*), 7.64 (d, 2H, *J* = 8 *Hz*), 7.68 (m, 5H, phenyl), 8.87 (s, 1H, NH), 11.10 (s, 1H, NH); ^13^C-NMR: *δ* = 117.1 (CN), 113.9, 118.7, 120.6, 124.0, 129.3, 130.6, 138.7, 142.4 (aromatic carbons), 155.3 (C-CN), 156.8 (C-NH_2_); MS, *m*/*z* (%), 312.1 (M^+^, 100), 77 (55); Anal. Calcd. for C_15_H_13_ClN_6_: C, 57.60; H, 4.19; Cl, 11.34; N, 26.87. Found: C, 57.58; H, 4.14; Cl, 11.29; N, 26.80. 

*3-Amino-2-p-tolylhydrazono-3-phenylhydrazonopropanenitrile* (**2b**). Yellow solid (68%), m.p. 148–150 °C; IR: *υ* = 3330 (br., NH_2_ and NH groups), 2181 (CN) 1630, 1620 (two C=N) cm^−1^; ^1^H-NMR: *δ* = 2.29 (s, 3H, CH_3_), 4.03 (s, 2H, NH_2_), 7.04 (d, 2H, *J* = 8 *Hz*), 7.46 (d, 2H, *J* = 8 *Hz*), 7.42 (m, 5H, phenyl), 9.67 (s, 1H, NH), 12.48 (s, 1H, NH); ^13^C-NMR: *δ* = 17.2 (CH_3_), 117.6 (CN), 116.1, 119.3, 119.8, 122.4, 124.2, 128.6, 131.1, 141.2 (aromatic carbons), 153.5 (C-CN), 154.8 (C-NH_2_); MS, *m*/*z* (%), 292.1 (M^+^, 100), 190.1 (52); Anal. Calcd. for C_16_H_16_N_6_: C, 65.74; H, 5.52; N, 28.75. Found: C, 65.47; H, 5.48; Cl, N, 28.70. 

### Reaction of ***2a,b*** with DMFDMA 

A mixture of **2a,b** (10 mmol) and DMFDMA (1.2 g, 10 mmol) was dissolved in xylene (50 mL) and the reaction mixture was refluxed for 8 h, then concentrated under reduced pressure to the half of its original volume, cooled and the solid product, so formed, was then filtered and recrystallized from ethanol. 

*2-p-Chlorophenyl-5-phenylhydrazono-2,5-dihydro-1,2,4-triazine-6-carbonitrile* (**5a**). Yellow solid (65%); m.p. 151–152 °C; IR: *υ* = 3350 (br., NH group), 2190.3 (CN) cm^−1^; ^1^H-NMR: *δ* = 7.02 (d, 2H, aryl o-protons), 7.52 (m, 7H, aromatic H), 9.67 (s, 1H, triazine H), 12.48 (s, 1H, NH); ^13^C-NMR: *δ* = 117.3 (CN), 114.8, 119.4, 121.3, 126.5, 127.9, 129.3, 138.4, 141.1 (aromatic carbons), 153.2 (C-CN), 154.7 (C-5, triazine ring), 158.6 (C-3, triazine ring); MS, *m*/*z* (%), 322.1 (M^+^, 100), 77.1 (32); Anal. Calcd. for C_16_H_11_ClN_6_: C, 59.54; H, 3.44; Cl, 10.98; N, 26.04. Found: C, 59.48; H, 3.40; Cl, 10.93; N, 25.97. 

*5-Phenylhydrazono-2-p-tolyl-2,5-dihydro-1,2,4-triazine-6-carbonitrile* (**5b**). Yellow solid (62%); m.p. 224–225 °C; IR: *υ* = 3345 (br., NH group), 2187 (CN) cm^−1^; ^1^H-NMR: *δ* = 1.63 (s, 3H, CH_3_), 6.85 (d, 2H, aryl o-protons), 7.43 (m, 7H, aromatic H), 8.76 (s, 1H, triazine H), 11.03 (s, 1H, NH); ^13^C-NMR: *δ* = 13.8 (CH_3_), 116.9 (CN), 116.4, 120.2, 121.8, 124.2, 128.2, 129.8, 137.1, 140.8 (aromatic carbons), 153.7 (C-CN), 155.4 (C-5, triazine ring), 157.5 (C-3, triazine ring); MS, *m*/*z* (%), 302.1 (M^+^, 100), 77.1 (83); Anal. Calcd. for C_17_H_14_N_6_ C, 67.54; H, 4.67; N, 27.80. Found: C, 67.51; H, 4.64; N, 27.73.

### Cyclization of phenylhydrazono-1,2,4-triazines 5a,b upon refluxing in AcOH

A mixture of **5a,b** (10 mmol) was dissolved in glacial acetic acid (30 mL) and the reaction was refluxed for 6 h while the reaction was followed to completion by TLC. The reaction mixture was neutralized with Na_2_CO_3_ solution whereby a solid product was formed. The solid was collected by filtration and recrystallized from ethanol. 

*2-p-Chlorophenyl-6-phenyl-2,6-dihydro-pyrazolo[3,4-e][1,2,4]triazin-7-one* (**6a**). Yellow solid (65%); m.p. 166–167 °C; IR: *υ* = 1689 (C=O) cm^−1^; ^1^H-NMR: *δ* = 6.62 (d, 2H, aryl o-protons), 7.34–7.98 (m, 7H, aromatic H), 7.67 (s, 1H, triazine H); ^13^C-NMR: *δ* = 114.7, 116.8, 119.1, 122.5, 128.7, 129.8, 134.1, 138.0, 139.6, 142.4 (aromatic carbons), 154.7 (C=O), 162.6 (C-3, triazine ring); MS, *m*/*z* (%), 323.1 (M^+^, 100), 225.1 (38), 77.1 (29); Anal. Calcd. for C_16_H_10_ClN_5_O: C, 59.36; H, 3.11; Cl, 10.95; N, 21.63. Found: C, 59.31; H, 3.04; Cl, 10.88; N, 21.56. 

*6-Phenyl-2-p-tolyl-2,6-dihydro-pyrazolo[3,4-e][1,2,4]triazin-7-one* (**6b**). Yellow solid (67%); m.p. 194–196 °C; IR: *υ* = 1681 (C=O) cm^−1^; ^1^H-NMR: *δ* = 1.98 (s, 3H, CH_3_), 6.81- 7.56 (m, 10H, aromatic protons); ^13^C-NMR: *δ* = 16.2 (CH_3_), 115.7, 119.0, 120.8, 122.7, 124.6, 128.9, 130.0, 138.2, 140.9, 142.4 (aromatic carbons), 154.6 (C=O), 158.3 (C-3, triazine ring); MS, *m*/*z* (%), 303.1 (M^+^, 100), 211.1 (46); Anal. Calcd. for C_17_H_13_N_5_O C, 67.32; H, 4.32; N, 23.09. Found: C, 67.31; H, 4.27; N, 23.02.

### Alternate route to ***6a,b*** by refluxing pyrazolones ***7a,b*** with DMFDMA (chemical evidence)

A mixture of **7a,b** (10 mmol) and DMFDMA (1.2 g, 10 mmol) was dissolved in dry xylene (50 mL) and the solution was refluxed for 8 h. The reaction mixture was triturated as usual and the solid product, so formed, was then filtered and recrystallized from ethanol. Elemental analysis and NMR spectra matched those of **6a,b**.

### Reaction of phenylhydrazono-1,2,4-triazines ***5a,b*** with hydroxylamine

A mixture of phenylhydrazono-1,2,4-triazine **5a,b** (10 mmol), hydroxylamine hydrochloride (0.69 g, 10 mmol) and sodium acetate anhydrous (3 g, 25 mmol) in ethanol (25 mL) was heated under reflux for 5 h. The reaction mixture was poured to water while a solid product was formed. The solid product, so formed, was then collected by filtration and recrystallized from ethanol to give **8a,b**. 

*2-p-Chlorophenyl-N′-hydroxy-5-phenylhydrazono-2,5-dihydro-1,2,4-triazine-6-carboximidamide* (**8a**). Yellow solid (60%); m.p. 218 °C (with decomposition); IR: *υ* = 3330 (br., OH and NH groups) cm^−1^; ^1^H- NMR: *δ* = 6.15 (s, 2H, NH_2_), 6.75-7.34 (m, 9H, aromatic H), 7.52 (s, 1H, triazine H), 9.37 (s, 1H, OH group), 11.65 (s, 1H, NH group); ^13^C-NMR: *δ* = 114.7, 119.2, 122.8, 128.2, 129.1, 132.9, 138.1, 139.6 (aromatic carbons), 155.2 (C-6, triazine ring), 156.3 (C-5, triazine ring), 156.9 (C-3, triazine ring), 158.6 (C=NOH); MS, *m*/*z* (%), 355.1 (M^+^, 100), 171.1 (87), 77.1 (75); Anal. Calcd. for C_16_H_14_ClN_7_O: C, 54.01; H, 3.97; Cl, 9.96; N, 27.56. Found: C, 53.95; H, 3.91; Cl, 9.89; N, 27.49. 

*N′-Hydroxy-5-phenylhydrazono-2-p-tolyl-2,5-dihydro-1,2,4-triazine-6-carboximidamide* (**8b**). Yellow solid (55%); m.p. 215 °C; IR: *υ* = 3320 (br., OH and NH groups) cm^−1^; ^1^H-NMR: *δ* = 2.24 (s, 3H, CH_3_), 5.57 (s, 2H, NH_2_), 6.52 (d, 2H, o-aryl protons), 7.06-7.94 (m, 7H, aromatic H), 7.64 (s, 1H, triazine H), 10.31 (s, 1H, OH group), 13.03 (s, 1H, NH group); ^13^C-NMR: *δ* = 18.1 (CH_3_), 115.2, 118.5, 122.8, 124.2, 126.3, 132.7, 134.2, 135.4 (aromatic carbons), 156.9 (C-6, triazine ring), 158.1 (C-5, triazine ring), 159.2 (C-3, triazine ring), 161.8 (C=NOH); MS, *m*/*z* (%), 335.1 (M^+^, 100), 105.1 (30); Anal. Calcd. for C_17_H_17_N_7_O: C, 60.88; H, 5.11; N, 29.24. Found: C, 60.84; H, 5.07; N, 29.18. 

### Cyclization of 1,2,4-triazine-6-carboximidamides ***8a,b*** upon refluxing with DMF

A solution of **8a,b** (10 mmol) in DMF (30 mL) was refluxed for 4 h. The reaction mixture was poured on HCl/ice mixture. The solid product, so formed, was then filtered and recrystallized from ethanol to give **10a,b**. 

*6-p-Chlorophenyl-2-phenyl-2,6-dihydro-[1,2,4]triazino[5,6-d][1,2,3]triazin-4-amine* (**10a**). Yellow solid (66%); m.p. 221–223 °C; IR: *υ* = 3350 (br., NH_2_ group) cm^−1^; ^1^H-NMR: *δ* = 6.14 (s, 2H, NH_2_), 7.44-7.8.04 (m, 9H, aromatic H), 9.51 (s, 1H, triazine H); ^13^C-NMR: *δ* = 115.4, 116.8, 119.0, 120.6, 123.8, 128.9, 139.0, 141.8 (aromatic carbons), 144.7 (C-6, 1,2,4-triazine ring), 146.4 (C-5, 1,2,4-triazine ring), 154.8 (C-NH_2_, 1,2,3-triazine), 157.2 (C-3, 1,2,4-triazine ring); MS, *m*/*z* (%), 337.1 (M^+^, 100), 171.0 (36), 77.1 (12); Anal. Calcd. for C_16_H_12_ClN_7_: C, 56.89; H, 3.58; Cl, 10.50; N, 29.03. Found: C, 56.84; H, 3.52; Cl, 10.45; N, 28.95. 

*2-Phenyl-6-p-tolyl-2,6-dihydro-[1,2,4]triazino[5,6-d][1,2,3]triazin-4-amine* (**10b**). Yellow solid (70%); m.p. 181–183 °C; IR: *υ* = 3340 (br., NH_2_ groups) cm^−1^; ^1^H-NMR: *δ* = 2.36 (s, 3H, CH_3_), 6.13 (s, 2H, NH_2_), 6.68 (d, 2H, o-aryl protons), 6.87-7.62 (m, 7H, aromatic H), 9.34 (s, 1H, 1,2,4-triazine H); ^13^C-NMR: *δ* = 17.8 (CH_3_), 114.6, 115.6, 123.2, 125.0, 128.3, 128.8, 136.9, 137.2 (aromatic carbons), 147.2 (C-6, 1,2,4-triazine), 148.8 (C-5, 1,2,4-triazine), 150.1 (C-NH_2_, 1,2,3-triazine), 157.5 (C-3, 1,2,4-triazine); MS, *m*/*z* (%), 317.1 (M^+^, 100), 77.1 (37); Anal. Calcd. for C_17_H_15_N_7_: C, 64.34; H, 4.76; N, 30.90. Found: C, 64.28; H, 4.72; N, 30.81. 

## Conclusions

Arylhydrazonomalononitriles proved to be versatile readily obtainable starting materials for the synthesis of pyrazolo[3,4-*e*][1,2,4]triazines and 1,2,4-triazino[5,6-*d*][1,2,3]triazines derivatives (the latter being a new ring system).
